# Exploiting Mitochondrial Dysfunction for Effective Elimination of Imatinib-Resistant Leukemic Cells

**DOI:** 10.1371/journal.pone.0021924

**Published:** 2011-07-18

**Authors:** Jérome Kluza, Manel Jendoubi, Caroline Ballot, Abir Dammak, Aurélie Jonneaux, Thierry Idziorek, Sami Joha, Véronique Dauphin, Myriam Malet-Martino, Stéphane Balayssac, Patrice Maboudou, Gilbert Briand, Pierre Formstecher, Bruno Quesnel, Philippe Marchetti

**Affiliations:** 1 Equipe 4 UMR 837 Inserm, Université de Lille II, Faculté de Médecine, Lille, France; 2 Equipe 3 UMR 837 and Institut de Recherche sur le Cancer de Lille, Lille, France; 3 Laboratoire de Synthèse et Physico-Chimie de Molécules d'Intérêt Biologique, UMR CNRS 5068 Université Paul Sabatier, Toulouse, France; 4 Centre de Bio-Pathologie, Plate-forme de Biothérapie, et Banque de Tissus, Centre Hospitalier Régional Universitaire, Lille, France; University of Medicine and Dentistry of New Jersey, United States of America

## Abstract

Challenges today concern chronic myeloid leukemia (CML) patients resistant to imatinib. There is growing evidence that imatinib-resistant leukemic cells present abnormal glucose metabolism but the impact on mitochondria has been neglected. Our work aimed to better understand and exploit the metabolic alterations of imatinib-resistant leukemic cells. Imatinib-resistant cells presented high glycolysis as compared to sensitive cells. Consistently, expression of key glycolytic enzymes, at least partly mediated by HIF-1α, was modified in imatinib-resistant cells suggesting that imatinib-resistant cells uncouple glycolytic flux from pyruvate oxidation. Interestingly, mitochondria of imatinib-resistant cells exhibited accumulation of TCA cycle intermediates, increased NADH and low oxygen consumption. These mitochondrial alterations due to the partial failure of ETC were further confirmed in leukemic cells isolated from some imatinib-resistant CML patients. As a consequence, mitochondria generated more ROS than those of imatinib-sensitive cells. This, in turn, resulted in increased death of imatinib-resistant leukemic cells following *in vitro* or *in vivo* treatment with the pro-oxidants, PEITC and Trisenox, in a syngeneic mouse tumor model. Conversely, inhibition of glycolysis caused derepression of respiration leading to lower cellular ROS. In conclusion, these findings indicate that imatinib-resistant leukemic cells have an unexpected mitochondrial dysfunction that could be exploited for selective therapeutic intervention.

## Introduction

Chronic myeloid leukaemia (CML) is provoked by the BCR-ABL translocation which leads to clonal myeloid cell expansion. The tyrosine kinase Bcr-Abl activates many intracellular signalling cascades conferring clear advantages for cancer cells. The apparition of the first competitive inhibitor of the Bcr-Abl tyrosine kinase, imatinib (Gleevec) in 2001 has revolutionized the prognosis of CML. In an attempt to improve the outcome of CML patients, second generation tyrosine kinase inhibitors (TKI) including dasatinib have been developed [1]. Unfortunately, development of resistance to imatinib is frequent and up to 28% of patients may have to stop imatinib because of secondary resistance. In addition, blasts from transformed CML are also mostly chemo-resistant, leaving no treatment option. Several mechanisms can explain the loss of imatinib sensitivity. Emergence of mutations within the ATP pocket of the Bcr-Abl kinase domain providing constitutive kinase activity plays an important role in imatinib resistance [2]. In humans, the most prevalent mutation E255K confers resistance to imatinib only, while others like T315I lead to multiple resistances including second-generation TKI [3]. However, imatinib resistance in CML is not necessarily caused by BCR-ABL mutations and CML patients who escape to imatinib therapy frequently express a non mutated form of BCR-ABL [4].

Therefore studies aimed to develop innovative strategies to overcome imatinib resistance and to eradicate CML are needed. Recently, the metabolic profile of Bcr-Abl (+) cells has attracted widespread interest. Activation of the PI3-kinase/Akt/mTOR pathway by Bcr-Abl contributes to the high glycolytic activity observed in Bcr-Abl positive cells [5], [6]. Expression of the Bcr-Abl kinase in haematopoietic cells stimulates increase in glucose uptake [7] through a PI3-kinase-dependent translocation of glucose transporters to the plasma membrane [8]. Besides, the constitutive activity of the serine/threonine kinase Akt has been shown to increase glucose consumption and lactate production in many cancer cells [9].

Imatinib, which causes the inhibition of the Bcr-Abl signalling cascade, controls the glucose flux by inhibition of the subcellular redistribution of the glucose transporter, GLUT1 [5] and also markedly affects metabolic organization of Bcr-Abl positive cells [6], [10]. At clinically relevant concentrations, imatinib strongly suppresses cytosolic glycolysis then increases the TCA cycle intermediates evoking a compensatory activation of mitochondrial function [10], [11]. In contrast, imatinib-resistant cells maintain highly elevated glycolysis irrespective of the treatment [12] suggesting that increased glucose metabolism participates to imatinib resistance.

Additionally, Bcr-Abl expressing cells harbour high levels of intracellular reactive oxygen species (ROS) and it has been speculated that increased glucose metabolism in these cells leads to ROS overproduction [13]. In Bcr-Abl expressing cells, mitochondria are a relevant source of ROS [13]. However, ROS derived from NADPH oxidase 4 also play a role in survival signalling in CML [14]. Inhibition of the antioxidant system with PEITC leads to further increase in ROS levels resulting in selective cell death of Bcr-Abl positive cells [15], [16]. Interestingly, PEITC can also promote ROS-mediated death of imatinib-resistant leukemia cells [16].

Since mitochondria are at the center for bioenergetics metabolism and ROS generation, we investigated the regulation of energy metabolism in imatinib-sensitive and -resistant leukemic cells focusing on mitochondrial functions. In this report, we provided a comprehensive analysis of the changes observed in the structure and function of mitochondria that occur in imatinib-resistant leukemic cells and demonstrated that exploiting the differential metabolic phenotype of imatinib-resistant cells presents opportunities for therapeutic action.

## Materials and Methods

### Chemicals

All chemicals were purchased from Sigma-Aldrich (St Louis, MO, USA) except 5,5′ ,6,6′ -tetrachloro-1,1′ ,3,3′ -tetraethylbenzimidazolyl- carbocyanine iodide (JC-1), MitoTrackerGreen (MTG) and Mitosox Red from Molecular Probes (Eugene, OR, USA).

### Cell culture conditions

From the murine DA1-3b cell line generated by stable transfection of bcr-abl [17], we established the clone DA1-3b/M2 by adding increasing concentrations of imatinib and dasatinib to the culture medium [18]. Acquired resistance of DA1-3b/M2 is mediated by composite T315I and E255K BCR-ABL mutations [18]. DA1-3b and DA1-3b/M2 were grown at 37°C under 5% CO_2_ in DMEM medium (Gibco-BRL, Life Technologies SARL, Cergy-Pontoise, France) containing 25 mM glucose supplemented with 10% fetal calf serum (Gibco-BRL), 50 U/ml penicillin, 50 µg/ml streptomycin, and 1 mM sodium pyruvate (Gibco-BRL). For siRNA inhibition of HIF-1α, DA1-3b/M2 cells were either transfected with 100 nM SiRNA HIF-1α (sc-35562, Santa Cruz Biotechnology Inc., Heidelberg, Germany) or 100 nM control non-targeting SiRNA (sc-37007) using lipofectamine 2000 (Life Technologies, Gaithersburg, MD, USA) according to the manufacturer's protocol. The CML-derived K562 cell line and its resistant derived sublines, K562-NI (resistant to nilotinib and imatinib), and K562-IM with acquired imatinib resistance) (kindly provided by Pr François-Xavier Mahon, CHU Bordeaux, France) were cultured in DMEM medium. K-562-IM and K-562-NI were characterized by the absence of mutation in the BCR-ABL kinase domain [19], [20]. Bone marrow samples were obtained from 4 CML patients resistant to imatinib. Normal CD34+ cells were obtained from bone marrow aspirates from healthy donors that gave informed consent in accordance with the Declaration of Helsinki. For *in vivo* experiments, DA1-3b/M2 cells were transfected with eGFP on the pVITRO plasmid (InvivoGen, California, USA) using the Amaxa Nucleofactor device (Amaxa, Köln, Germany), with a Cell Line specific Nucleofector kit V (program O17) according to the manufacturer's instructions. After 48 h, cells were selected in medium containing Blasticidin at 20 µg/ml (InvivoGen) for up to 12 days.

### Ethics Statement

Informed signed consent for cell analysis was obtained from each patient and donor included and the study was approved by our institutional ethical committee (CPP Lille). All animal experiments were performed as approved by the Animal Care Ethical Committee CEEA.NPDC (Agreement # AF-03-2008).

### Glucose and lactate measurements

Glucose and lactate were measured in the extracellular medium using a SYNCHRON LX20 Clinical system (Beckman Coulter, Fullerton, CA USA).

### Cytofluorometric analysis

The selective detection of superoxide in the mitochondria of cancer cells was determined using the MitoSox Red probe following current protocols [21] and analyzed on a FACS Canto cytofluorometer equipped with the FACS-Diva research software (Beckton Dickinson). The inner mitochondrial transmembrane potential (ΔΨ_m_), was determined with JC-1 [22]. Results were the ratio between the fluorescence of sample cells and the fluorescence obtained after incubation of cells with the mitochondrial uncoupler, ClCCP. For mitochondrial mass, cells were incubated with 50 nM MTG as described [22]. Assessment of cell death was performed using the protocol outlined in the FITC-Annexin kit (BD Pharmingen, San Diego, CA, USA).

### Real-time Quantitative RT-PCR

Total RNAs were isolated using the RNAzol B method (WAK-Chemie Medical GmBH, Germany) according to manufacturer's instructions. cDNAs were synthesized with the High Capacity Transcription Kit (Applied Biosystems, CA, USA) from 1 µg of total RNA. For a list of primers, see Material and Methods S1. Real-time PCR was performed with the cDNA samples and Express SYBR green ER Supermixes (Invitrogen, Carlsbad, CA, USA) using a cycling protocol optimized for Lightcycler 480 detector (Roche Applied Science, Manheim Germany) as we described elsewhere [23]. All samples were amplified in triplicates. The transcripts level in each sample was normalized to that of 18S rRNA. The relative expression of target mRNAs in DA1-3b/M2 cells was compared with the expression in DA1-3b using the Pfafll method with a fold change criteria of 1.5 [24].

### NMR analysis

The frozen cell pellet (15×10^6^ cells) was extracted according to Beckonert's procedure [25] with CHCl_3_/MeOH/H_2_O (2∶2∶1.425 (v/v/v)). The lyophilisate was dissolved in 550 µL of D2O (Eurisotop, France) and 5 µL of a 5 mM solution of TSP (sodium 2,2,3,3-tetradeutero-3-trimethylsilylpropionate) (Sigma-Aldrich) were added as internal chemical shift and quantification reference. 1H NMR spectra were recorded on a Bruker Avance spectrometer. All 1H experiments were acquired at 298K using a classical 1D pulse sequence (relaxation delay-pulse-acquisition) with a 2.0 s pre-saturation pulse for water suppression and a repetition time of 6.1 s. A flip angle of 30° was employed with 32K data points for acquisition over a spectral width of 10 ppm, and 1024 scans were collected. Data were processed using Bruker TopSpin software 2.1 with one level of zero-filling, and Fourier transformation after multiplying FIDs by an exponential line-broadening function of 0.3 Hz. The concentrations of metabolites were calculated by comparing the expanded areas of their respective NMR signals with that of the internal standard for quantification TSP. The areas were determined by automatic integration using the KnowItAll® software.

### Immunoblot analysis

Cells (3×10^6^ for each sample) were resuspended in extraction buffer and lysates were prepared as described previously [26]. For analysis of OXPHOS complexes, 25 µg proteins were separated on a 4–12% SDS-PAGE denaturing gel and transferred to nitrocellulose membrane (Amersham BioSciences, Piscataway, NJ). Equivalent loading was checked by actin staining. Membranes were blocked in 5% milk in TBST buffer then probed with a premixed cocktail of five monoclonal antibodies (total OXPHOS antibody cocktail kit, MitoSciences, 1∶200) specifically recognizing CI subunit NDUFB8 (MS105), CII-30 kDa (MS203), CIII-Core protein 2 (MS304) CIV subunit I (MS404) and CV alpha subunit (MS507). For analysis of glycolysis-related proteins, 20 µg proteins were subjected to SDS-PAGE. Membranes were blocked in 10% powdered milk in TBS Tween 0.05% for 1 h at room temperature and then incubated with primary antibodies specific for SLC2A1 (1∶200, Santa Cruz, H-43), SLC2A6 (1∶500, Santa Cruz, M-16), Pdk3 (1∶500, Santa Cruz, RR-21), Lamin A/C(1∶200, Santa Cruz, 636), Hk2 (1∶500, Cell Signaling, C64G5), Pfkp (1∶200, Cell Signaling), AldoA (1∶200, Cell Signaling). Secondary horseradish peroxidase–conjugated antibodies (BioRAD) were used at 1∶2,000 for 1 h at room temperature, and detection was carried out by enhanced chemiluminescence. Anti-actin Ab (1∶5,000, Sigma) was used for standardization of protein loading. For HIF-1α, nuclear fractions (30 µg), prepared with a commercial kit (nuclear fractionation kit from BioVision), were analyzed using anti- HIF-1α monoclonal Ab (1∶500, Santa Cruz, H-206). Anti lamin A/C Ab (1∶200, Santa Cruz, 636) was used to check for equal loading.

### Pyridine nucleotide and ATP measurements

We analyzed the pyridine nucleotides (NAD+/NADH ratio) in whole cell lysates and the level of NADH in mitochondria isolated from cells with the NAD^+^/NADH Quantification assay kit according to manufacturer's instructions (BioVision, Mountain View, CA). For ATP determination, the Celltiter Glow Assay kit (Promega) was used according to the manufacturer's procedures.

### Measurement of oxygen consumption

Cells were suspended at 2×10^6^ cells/ml in cell culture medium. Oxygen consumption was monitored over time with a polarographic oxygen electrode (Oxygraph-2k, Oroboros Instruments, Innsbruck, Austria) as described [27]. Cell viability was assessed by propidium iodide staining at the end of this experiment. In respiration studies using non-permeable substrates of respiratory chain, cells were permeabilized with digitonine (50 µg/ml final concentration) in the oxygraph chamber.

### Analysis of mitochondrial respiratory chain complex enzyme activities

Mitochondria were purified from cells following standard protocols [26]. Isolated mitochondria (1 mg/ml) were suspended in buffer containing 75 mM saccharose, 225 mM mannitol, 0.1 mM EDTA and 10 mM Tris-HCl, pH 7.2 and incubated on ice. Samples were incubated at 37°C for 15 minutes. Enzyme activities were immediately assayed at 30°C spectrophotometrically using a SAFAS UVMc2 spectrophotometer (Safas Monaco) following standard procedures [28].

### NADPH oxidase assay

NADPH oxidase activity was measured in membrane fractions by lucigenin chemiluminescence. 5 µg of proteins were incubated with 50 µM lucigenin in HBSS 1×. The reaction was started by adding 100 µM NADPH then relative light units (RLU) of chemiluminescence were read in a 96-well luminometer (Mithras, LB940, Berthold Technologies, Thoiry, France). Photon emission was measured every 15 s for the initial 5 min corresponding to the linear portion of luminescence generation. The background chemiluminescence of the buffer was always subtracted from each reading before data calculations. There was no measurable activity in the absence of NADPH.

### Transmission electron microscopy

Cells were prepared for transmission electron microscopy as described previously [22] .

### *In vivo* study

Seven to 8-week-old C3H/HeOUJ female mice (Charles River Laboratories) were injected i.p. with 1×10^6^ GFP-transfected DA-13b/M2 cells. Treatment started 24 h after cell inoculation with i.p. injection of vehicle, Trisenox (As2O3; 4 mg/kg five times a week) or PEITC (PhenylEthyl IsothioCyanate); 50 mg/kg five times a week. Drug injections were terminated after one month and mice were monitored daily for signs of tumor growth and shaggy hair. Moribund animals were sacrificed and spleens were removed. Residual M2-GFP cells were immediately analyzed by flow cytometry for viability and ROS production.

### Statistical analysis

The student's *t-test* was used to compare data sets using GraphPad Prism® version 4.00 (GraphPad Software, San Diego, CA, USA). Statistical significance was set at P<0.05. Kaplan-Meier curve comparison was performed with the log-rank test.

## Results

### Glucose metabolism in imatinib-sensitive and imatinib-resistant leukemic cells

**Figure 1:**
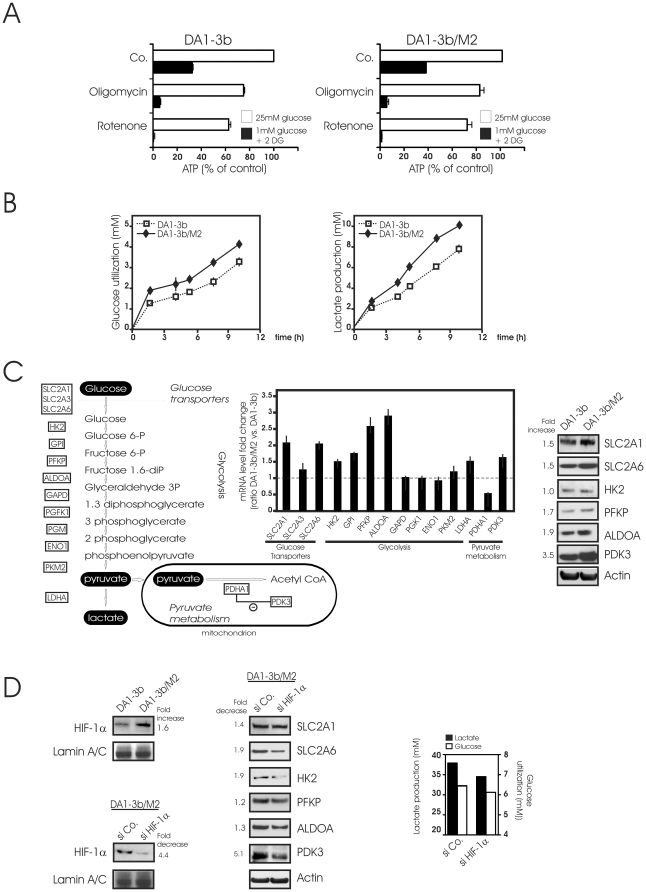
Characterization of glucose metabolism in imatinib-sensitive and -resistant cells. (A) DA1-3b (imatimib-sensitive) and DA1-3b/M2 (imatinib-resistant) leukemic cells were cultured in DMEM (containing 25 mM of glucose) (□) or in medium containing 1 mM glucose and 10 mM 2-Deoxyglucose (2-DG) (▪) for 18 h, then treated with 1 µg/ml oligomycin or 1 µM rotenone for 4 h. Cells were then collected for total ATP measurement as described in Materials and Methods. Data shown are means +/− SD of four separate cell preparations. (B) Exponentially growing DA1-3b and DA1-3b/M2 cells were resuspended in fresh DMEM medium at a density of 2×10^6^ cells/ml. At indicated times, glucose (left) and lactate (right) were measured in the supernatants. Data are means +/− SD of three independent experiments (C) (left) Schematic overview of glucose metabolism to point out the position of studied genes in metabolism. (middle) Quantitative PCR analysis of relative transcript levels of glucose transporters, glycolytic enzymes and enzymes related to lactate metabolism in DA1-3b/M2 cells compared with DA1-3b cells. Data are means +/− SD of four independent experiments. (right) Immunoblot analysis of protein abundance in DA1-3b and DA1-3b/M2 cells. Densitometric values of proteins are normalized on the basis of actin expression and expressed as fold induction in DA1-3b/M2 cells over DA1-3b cells (n = 2). (D) (left) Expression of HIF-1α protein in DA1-3b and DA1-3b/M2 cells. Nuclear extracts were analyzed by immunoblotting against HIF-1α. Lamin A/C was used as a loading control. Alternatively, DA1-3b/M2 cells were treated with either HIF-1α siRNA or control non-targeting siRNA. Knock down of HIF-1α was confirmed by immunoblot at 48 h post transfection. Data are representative of 3 different experiments. (middle). The effect of HIF-1α knock-down in DA-13b/M2 cells on expression of glycolysis-related proteins. Protein expression was measured by immunoblot densitometry and normalized to actin. The values in HIF-1α silenced DA1-3b/M2 cells are reported as the fold decrease over control (n = 2). (right) Effects of loss of HIF-1α expression in DA-13b/M2 cells on glucose utilization and lactate production. Exponentially growing DA1-3b/M2 cells transfected with siControl or si HIF-1α were incubated in fresh DMEM medium at a density of 2×10^6^ cells/ml. At 24 h, glucose and lactate were measured in the supernatants. Data are means of two independent experiments.

We have previously established a cellular model to imitate the process of imatinib resistance in patients [18]. From the parental Bcr-Abl (+) leukemic cell line DA1-3b which is sensitive to tyrosine kinase inhibitors, we have generated the imatinib and dasatinib resistant cell line, DA1-3b/M2, carrying the most frequent Bcr-Abl mutations (T315I and E255K) associated with resistance in patients [18]. In normal culture with DMEM containing 25 mM glucose, DA1-3b and DA1-3b/M2 cell lines had a similar ATP content (0.640+/−0.04 vs. 0.644+/−0.10 nmol/10^6^ cells; n = 3). Incubation of DA1-3b and DA1-3b/M2 cells in low glucose medium with the inhibitor of glycolysis, 2-DG, yielded a drastic depletion of more than 60% in cellular ATP level. By comparison, suppression of the mitochondrial oxidative phosphorylation with the respiratory inhibitor, rotenone or the phosphorylation inhibitor oligomycin did not exert a major inhibitory effect on cellular ATP production (Fig. 1A). Additionally, both cell lines maintained, under normoxic conditions, avid glucose consumption with concomitant high lactate production. When compared with DA1-3b, DA1-3b/M2 cells were more active in glucose utilization and produced more lactate (Fig. 1B). Consistently, we identified by quantitative real-time PCR 7 genes involved in glycolysis which were specifically up-regulated in DA1-3b/M2 cells. Glucose transporter genes (*SCL2A1*, *SCL2A6*), genes (*HKII*, *GPI*, *PFKP*, *ALDOA*) encoding enzymes involved in the first four steps of the glycolysis were up-regulated in DA1-3b/M2 cells. Similarly, up-regulation of *LDHA* was observed in DA1-3b/M2. In addition, we assumed an impaired oxidative metabolism of pyruvate in DA1-3b/M2 mitochondria since *PDH* expression was decreased with a concomitant increase in *PDK3* as compared to DA1-3b cells. In line with qPCR results, the levels of some important glycolysis-related proteins were up-regulated in DA1-3b/M2 cells as determined by immunoblot (Fig. 1C). These experiments were repeated using the human CML-derived K562 cell line and its resistant derived sublines (K562-IM, K562-NI) with similar outcomes (FigS1). Since HIF-1α is a master transcriptional regulator of glucose metabolism in cancer cells, we investigated the role of HIF-1α in glycolysis in imatinib-resistant cells. Under normoxic condition, expression of HIF-1α was found elevated in imatinib-resistant DA1-3b/M2 cells as compared to sensitive cells (Fig. 1D, left). To determine whether HIF-1α could be contributing to glycolysis in these cells, DA1-3b/M2 cells were transfected with siRNA targeting HIF-1α which consistently decreased its expression by ∼75% (Fig. 1D, left). In these conditions, expression of several glycolysis-related proteins was decreased in HIF-1α siRNA transfected DA1-3b/M2 cells compared to control cells (Fig. 1D, middle). Consistently, glucose utilization and lactate production were also decreased in HIF-1α silenced cells (Fig. 1D, right). Thus, imatinib-resistant leukemic cells present increased glycolytic rate and shift to lactate production as compared to their sensitive counterparts. The highly glycolytic phenotype of imatinib-resistant leukemic cells was partially dependent on HIF-1α activity.

### Mitochondrial characteristics of imatinib-sensitive and imatinib-resistant leukemic cells

**Figure 2:**
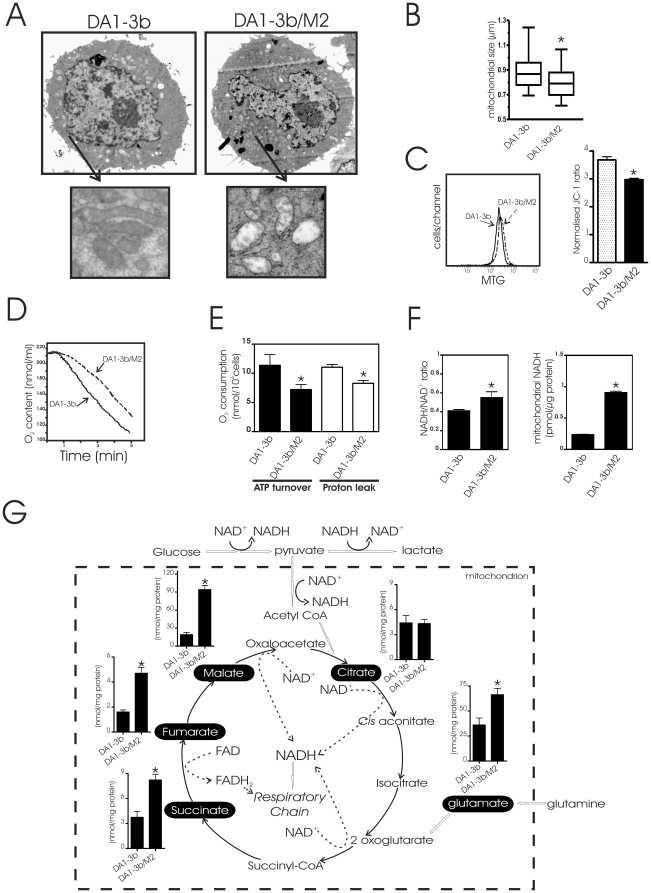
Characterization of mitochondrial alterations in imatinib-resistant cells. (A) Transmission electron microscopic images of DA1-3b (imatimib-sensitive), DA1-3b/M2 (imatinib-resistant) cells. Typical representative morphologic features of cells (magnification, ×7,000) and mitochondria (magnification, ×20,000); (B) Size of mitochondria per DA1-3b cells or DA1-3b/M2 cells. A total of 500 cells were examined in transmission electronic microscopy and mitochondrial size were evaluated. Values are medians (horizontal bars) with 25–75% interquartile ranges (boxes) and minimum–maximum values (I) (*P = 0.02 between DA1-3b and DA1-3b/M2 cells); (C) (left) Representative flow cytometric measurement of mitochondrial mass. DA1-3b and DA1-3b/M2 cells were labeled with the MTG fluorescent dye. (right) Flow cytometric measurement of ΔΨ_m_. DA1-3b and DA1-3b/M2 cells were labeled with JC-1. JC-1 fluorescence (mean fluorescence intensity) ratio is normalized to that of depolarized cells (cells incubated with 20 µM ClCCP used as a positive control for loss of ΔΨm). Values are means ± S.D of five measurements; (D) Comparison of oxygen consumption in DA1-3b and DA1-3b/M2 cells. DA1-3b or DA1-3b/M2 cells (2×10^6^/ml) in DMEM were added to a closed chamber with an oxygen electrode at 37°C and oxygen levels were monitored over time. Data are representative of five independent experiments; (E) Proportions of mitochondrial oxygen consumption due to proton leak and ATP turnover reactions in DA1-3b and DA1-3b/M2 cells. Proton leak corresponds to the respiration that is not modified by the inhibitor of the ATP synthase, oligomycin whereas ATP turnover constitutes the respiration that is inhibited by oligomycin. Oxygen consumption was determined as in (D). Data are means +/− SD of three separate experiments; (F) Determination of cytoplasmic NADH/NAD ratio (left) and mitochondrial NADH in DA1-3b and DA1-3b/M2 cells. Data are means +/− SD of three independent experiments in triplicates; (G) Quantified levels of mitochondrial TCA cycle related metabolites in DA1-3b and DA1-3b/M2. Metabolites detected (black boxes) are represented on a comprehensive metabolic pathway map. Results are means +/− SD of fifteen ^1^H NMR spectra of the metabolome for each of the individual cell cultures.

Since imatinib-resistant cells present increased glycolysis, we suspected that DA1-3b/M2 cells have defective mitochondria. Transmission electron microscopy revealed that mitochondria in DA1-3b/M2 cells were smaller, with less electron-dense matrix (Fig. 2A and B), a feature already observed in leukemic cells with mitochondrial defects [29]. There was no difference in mitochondrial mass as detected by MTG fluorescence (Fig. 2C) and no difference in the number of mitochondria between DA1-3b and DA1-3b/M2 cells (not shown). We observed that DA1-3b/M2 cells had a slight lower ΔΨm than DA1-3b cells (Fig. 2C). Interestingly, DA1-3b/M2 cells in culture medium exhibited a significantly lower oxygen consumption rate than DA1-3b (Fig. 2D). The decrease in oxygen consumption rates can be explained by a decrease in ATP turnover (respiration coupled to ATP synthesis) as well as a decrease in proton leak (uncoupled respiration) (Fig. 2E). In a similar manner, mitochondrial dysfunction was associated with imatinib resistance of human CML K562 cells (Fig. S2). We found an increase in NADH/NAD+ ratio and mitochondrial NADH concentration in DA1-3b/M2 cells (Fig. 2F). Consistently, when we studied the tricarboxylic acid (TCA) cycle that connects glucose metabolism to oxidative phosphorylation, an accumulation of TCA intermediates (succinate, fumarate and malate) and elevated level of glutamate, a major respiratory substrate of cancer cells [30], were observed in DA1-3b/M2 cells in comparison with DA1-3b (Fig. 2G). Thus, accumulation of TCA intermediates, which correlated to NADH increase, may indicate alterations of the electron transport chain (ETC) in mitochondria of imatinib-resistant cells.

### Mitochondrial respiration defects in imatinib-resistant leukemic cells

**Figure 3:**
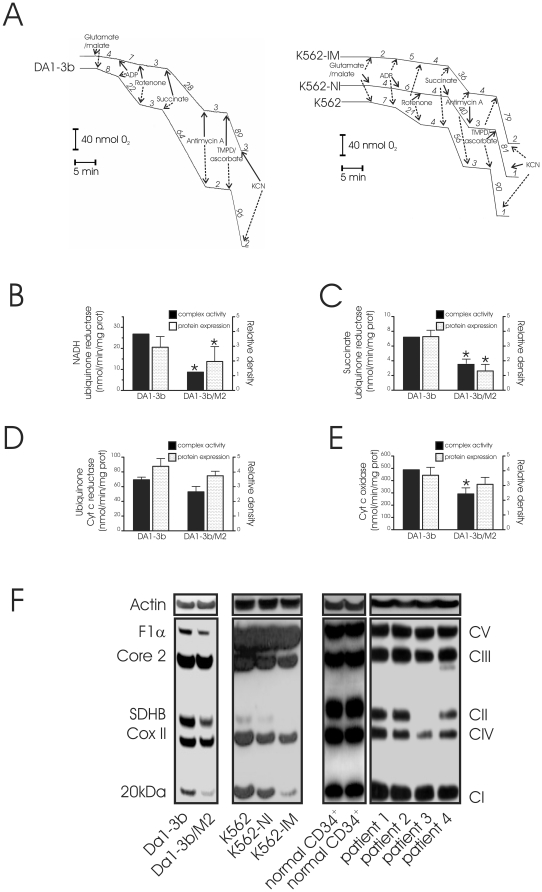
Impairment of the mitochondrial electron chain complex activity in imatinib resistant cells. (A) Traces of oxygen consumption of digitonin-permeabilized DA1-3b vs. DA1-3b/M2 cells (left), and K562 vs. K562-IM, K562-NI cells (right). Respiration in response to complex I (glutamate+malate, 5 mM each) or complex II (succinate, 5 mM) substrates was determined in the presence or absence of 1 mM ADP. Mitochondrial substrates, N,N,N′,N′-tetramethyl p-phenylenediamine (TMPD 0.5 mM) + ascorbate (1 mM) and respiratory inhibitors, rotenone (2 µM), antimycin A (1 µM) and potassium cyanide (KCN, 100 µM) were used as indicated: As expected, KCN used as control, blocked mitochondrial respiration. Values for oxygen consumption are represented on the curves as nmol oxygen/min; One representative experiment of five performed (B–E) Mitochondrial respiratory chain complex activities (left axes) and protein expression levels of complexes (right axes) in mitochondria isolated from DA1-3b and DA1-3b/M2 cells. (B) NADH ubiquinone reductase (Complex I), (C) Succinate cytochrome c reductase (Complex II+III), (D) Ubiquinone cytochrome c reductase (Complex III) and (E) cytochrome c oxidase (Complex IV activity) activities were measured by spectrophotometry as described in Material and Methods. Rotenone, antimycin A and sodium azide, (prototypic inhibitors of complex activities) were used as control. Quantitative densitometric analysis of mitochondrial repiratory chain proteins was evaluated on Western blot as depicted in (F). The results are expressed as a ratio of actin density following reprobing of the membrane. Results are representative of three independent experiments. (*P<0.05 between DA1-3b and DA1-3b/M2 cells); (F) Western blot analysis of mitochondrial respiratory chain complex proteins such as complex I (20 kDa subunit), complex II (SDHB), III (core 2 protein), IV (Cox II) and V (F1α ATPase). Protein expression patterns were assessed in DA1-3b and DA1-3b/M2 cells, in human K562 (sensitive to imatinib), K562-NI (resistant to nilotinib and imatinib) and K562-IM (resistant to imatinib) CML cell lines, in normal CD34+ cells (2 separate specimen) and in cells isolated from four CML patients. BCR-ABL (+) CML cells were isolated from 4 patients: one patient with primary resistance to imatinib (patient 1), two patients with secondary resistance to imatinib in accelerated/blastic phase (patients 3 and 4) of the disease and one patient (Patient 2) had only a partial response.

To analyze the loss of integrity of the ETC complexes, we measured substrate-driven oxygen consumption in digitonin-permeabilized cells (Fig. 3A). Compared with DA1-3b cells, respiration in DA1-3b/M2 cells with complex I- (glutamate/malate), or complex II-linked substrate (succinate), was significantly reduced both during state 3 (in the presence of ADP) and state 4 (without ADP). When we investigated electron flow post-cytochrome c to complex IV by using TMPD/ascorbate and antimycin A (to block complex III), DA1-3b/M2 showed a slightly lower rate of oxygen consumption (Fig. 3A, left part). As with murine cells, human imatinib-resistant cell lines, K562-IM and K562-NI, displayed decreased substrate-dependent respiration rate in comparison with imatinib sensitive K562 cells (Fig. 3A, right part).

To confirm these results, we measured the activities of respiratory enzymes in mitochondria isolated from both cell lines (Fig. 3B–E). NADH dehydrogenase (complex I) showed the highest reduction of activity in DA1-3b/M2 as compared with DA1-3b cells (3-fold lower, p<0.01). Mitochondria isolated from DA1-3b/M2 cell also displayed a significant reduction in the activities of succinate ubiquinone reductase (Complex II; 2-fold lower, p<0.05), and cytochrome c oxidase (Complex IV; approximatively 1.5-fold lower, p<0.05). Quinol cytochrome c reductase (QCCR, complex III) activity appeared much smaller in DA1-3b/M2 but was not statistically significant. We next analyzed components of mitochondrial ETC by Western blotting using a cocktail of monoclonal antibodies optimized for the detection of one subunit of each of the five complexes [31]. In further support of a reduction in mitochondrial functions, proteins of complexes I and II were significantly less abundant in DA1-3b/M2 cells than in DA1-3b cells, complexes IV and V appeared less expressed in DA1-3b/M2 cells but failed to reach statistical significance, complex III remained similar in both cell lines (Fig. 3F). Reduction in protein expression was correlated with the lower activities of mitochondrial complexes found in DA1-3b/M2 (Fig. 3B–E) suggesting that the corresponding complex failed to assemble properly. DA1-3b/M2 cells, which were recovered *ex vivo* from injected mice, retained the same expression of mitochondrial complexes (not shown). Moreover, two additional imatinib-resistant cell lines (K562-NI and K562-IM) had lower expression of most of the ETC proteins compared with their sensitive counterparts (K562) (Fig. 3F). In comparison with normal CD34 + cells, no changes in ETC protein expression was found in CML leukocytes isolated from patient with primary resistance to imatinib (patient 1) or from patient with partial response (patient 2) (Fig. 3F and Table S1). Conversely, reduction in ETC protein expression was evident in CML leukocytes isolated from two imatinib-resistant patients in the accelerated/blastic phase of the disease (patients 3 and 4, Fig. 3F and Table S1). Thus, mitochondrial alterations observed in DA1-3b/M2 cells were also found in additional imatinib-resistant leukemic cell lines as well as in samples from imatinib-resistant patients in blast crises.

### Influence of energy substrates on mitochondrial function in imatinib-resistant leukemic cells

**Figure 4:**
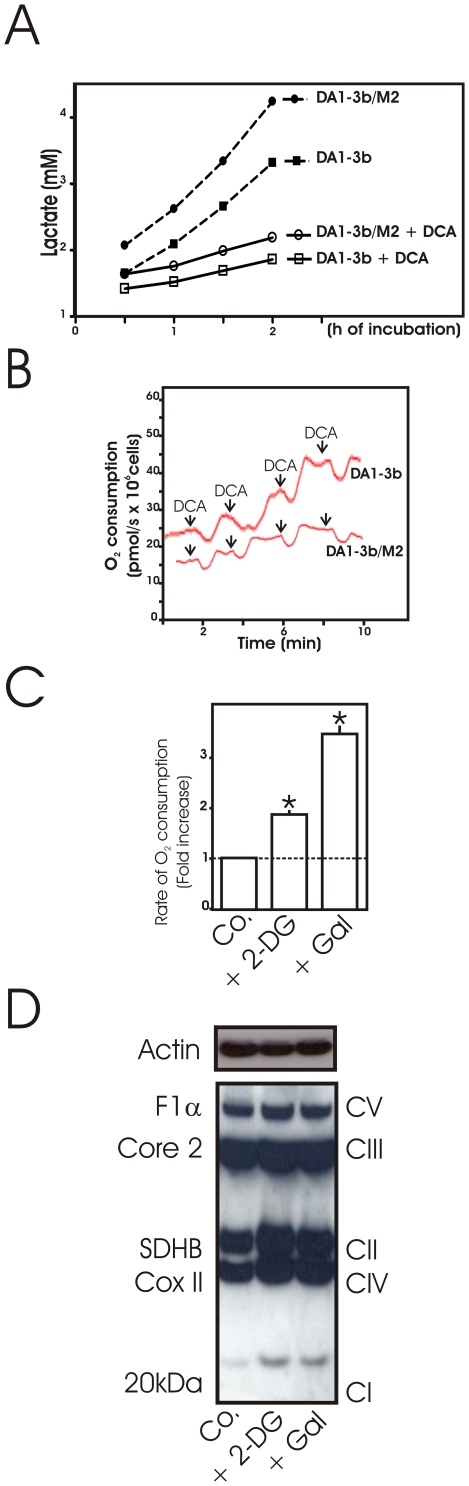
Regulation of mitochondrial dysfunction in imatinib resistant cells. (A) Effect of dichloroactetate (DCA) on lactate production. DA1-3b and DA1-3b/M2 cells were incubated in the presence of 6 mM DCA then at the indicated time points, lactate production was determined. Values are means of two independent experiments in triplicates; (B) The oxygen consumption was measured as described in Materials and Methods following the sequential addition of DCA (1.5 mM every 2 min, arrows) in DA1-3b and DA1-3b/M2 cells. Typical result out of two independent experiments; (C) Whole-cell oxygen consumption measurements in DA1-3b/M2 cells treated with 10 mM 2-DG for 18 h or grown in galactose DMEM medium for 48 h relative to untreated cells (Control, Co.). Data are means +/− SD of four independent experiments in duplicate; * indicates p<0.05 compared to control (D) Typical western blot analysis of mitochondrial respiratory chain complex proteins in DA1-3b/M2 cells treated as in (C). Actin was used as a loading control. Data are representative of three independent experiments.

One possible explanation for mitochondrial dysfunction in imatinib-resistant cells is that metabolic flux of pyruvate is rerouted through LDH reducing mitochondrial oxidation of pyruvate thus decreasing mitochondrial respiration. Dichloroacetate (DCA), an inhibitor of PDK, increases flux of pyruvate into mitochondria [32]. As expected from this effect, lactate levels in the culture medium of the DCA-treated cells decreased (Fig. 4A). In parallel, activating mitochondria by DCA increased oxygen consumption in both cell lines until saturation occurred (Fig. 4B). Notably, DCA-induced mitochondrial activation was less pronounced in DA1-3b/M2 even at saturable doses suggesting that ETC defects persist in DA1-3b/M2 mitochondria when oxidative phosphorylation is forced. Consistent with this result, treatment of DA1-3b/M2 cells with DCA was unable to restore the expression of ETC complexes (not shown).

One hallmark of high dividing cells is a glucose-induced repression of oxidative metabolism decreasing mitochondrial respiration [31]. To get insight into this possibility, we assessed the impact of glucose deprivation on mitochondrial respiration in DA1-3b/M2 cells (Fig. 4C). Compared to cells cultured in glucose medium, respiration rates were increased in galactose-grown DA1-3b/M2 cells and in cells incubated with 2-DG. As predicted by the aforementioned results, these culture conditions stimulated the expression of ETC proteins (Fig. 4D).

### Mitochondrial ROS production in imatinib-sensitive and imatinib-resistant leukemic cells

**Figure 5:**
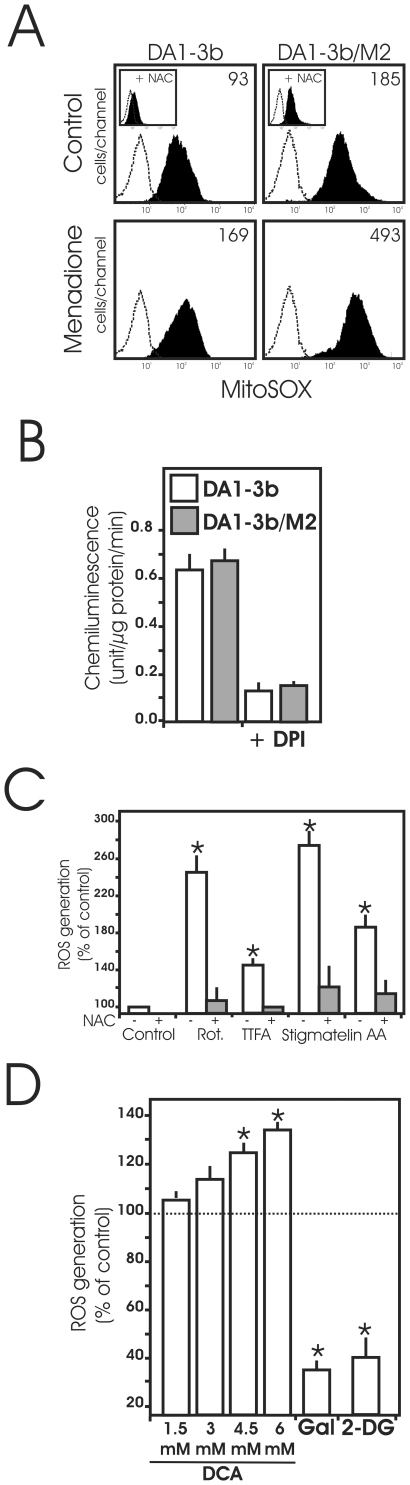
Mitochondrial ROS production in imatinib- sensitive and -resistant cell lines. (A) Cytofluorometric analysis of mitochondrial ROS production in DA1-3b and DA1-3b/M2. Cells were kept untreated (Control) or treated with the mitochondrial redox cycling promoter menadione (100 µm, 1 h) used as positive control then labeled with the fluorescent probe MitoSox as described in Materials and Methods. Alternatively, cells were treated with menadione in the presence of 10 mM NAC (inset). Data represent typical results of one out of three independent experiments; (B) NADPH oxidase activity in the plasma membrane of DA1-3b and DA1-3b/M2 cells. When indicated, cells were treated with the flavoprotein inhibitor diphenyleneiodonium (DPI), used as control. Lucigenin chemiluninescence assay was used as described in Material and methods. Results are means +/− SD of two independent experiments carried out in triplicates; (C) Effects of various inhibitors of the mitochondrial respiratory chain on ROS production in DA1-3b cells. Cells were kept untreated (Control) or treated with 1 µM rotenone (rot, a know inhibitor of mitochondrial electron transport complex I), 1 µM 2-thenoyltrifluoroacetone (TTFA, a conventional complex II inhibitor), 1 µM stigmatellin or 1 µM antimycin A (AA, two specific inhibitors of mitochondrial complex III) in the presence or absence of 10 mM N-acetyl cystein (NAC) for 18 h then the levels of ROS were determined by flow cytometry after Mitosox staining. Inhibitors were used at sub-toxic concentrations which induce moderate inhibition of cell respiration (around 20%). Data are means +/− SD of four independent experiments; * indicate significant differences from untreated cells at p<0.05 (D) DA1-3b/M2 cells were incubated in the presence of the indicated doses of DCA for 1 h, or with 10 mM 2-DG for 18 h, or grown in galactose medium for 48 h then the levels of ROS were determined by flow cytometry after Mitosox staining. Data are means +/− SD of four independent experiments. * indicate p<0.05 compared to control.

Transformation of cells by Bcr-Abl increases ROS production [33]. We considered the possibility that imatinib-resistant could exhibit higher level of ROS. We subsequently tested the steady-state levels of mitochondrial ROS in imatinib-resistant cells by measuring oxidation of the MitoSox Red (Fig. 5A and Fig. S3). Mitochondrial ROS were higher in DA1-3b/M2 than in DA1-3b cells and this increase was efficiently blocked by incubation with the thiol containing antioxidant N-acetyl cystein (NAC). As shown in Figure 5A, menadione, a generator of mitochondrial free radicals, stimulated the formation of ROS more efficiently in DA1-3b/M2 than in DA1-3b cells. The same results were obtained in human imatinib-resistant K562 cells (Fig. S3A) and when we used the superoxide sensitive fluorochrome Hydroethydine instead of MitoSox (not shown). As assessed by H-NMR, glutathione, the major component of cellular antioxidant barrier, was significantly lowered by 30% in DA1-3b/M2 cells relative to DA1-3b cells (21.8+/−1.9 vs. 30.8+/−3.4 p<0.001). Since it has been shown that ROS can be produced by activation of NADPH oxidase in Bcr-Abl expressing cells [14], we determined NADPH oxidase activity in the membrane of leukemic cells (Fig. 5B, right). In both cell lines, NADPH oxidase activity was present in the membrane fractions and was almost completely prevented by DPI, a specific inhibitor of flavoproteins. However, no difference in NADPH oxidase activity between imatinib-sensitive and -resistant cells was observed. Thus, unlike mitochondria, NADPH oxidase was not the major source of the increased ROS production observed in imatinib-resistant cells.

We assumed that mitochondrial alterations are likely to enhance ROS production. To test this hypothesis, we incubated DA1-3b cells with various mitochondrial respiration inhibitors before MitoSox Red staining (Fig. 5C). Interference with any complex of the electron transport chain led to ROS production that was prevented by pre-treatment of cells with NAC. In accord with this result, restoration of an intact ETC in DA1-3b/M2 cells after treatment with 2-DG or galactose (Fig. 4B) completely abolished the generation of ROS (Fig. 5D). Conversely, activation of mitochondria with persistent ETC defects by DCA significantly increased the level of ROS in DA1/3b-M2 cells. This underscores the strict correlation between mitochondrial dysfunction and ROS production indicating that the partial failure of ETC can be responsible for the excess of mitochondrial ROS in DA1-3b/M2 cells.

### Exploiting mitochondrial oxidative stress to kill imatinib-resistant cells

**Figure 6:**
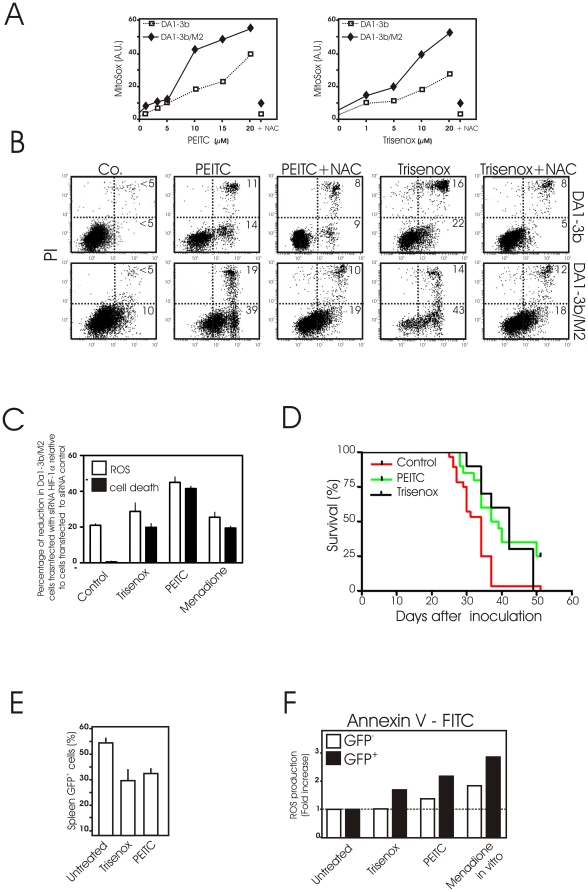
*In vitro* and *in vivo* antitumor activity of PEITC and Trisenox in imatinib resistant cells. (A) DA1-3b and DA1-3b/M2 cells were treated with 1, 3, 5, 10, 15 and 20 µM of the pro-oxidant phenethyl isothiocyanate (PEITC) for 18 h (left) or with 1, 5, 10 and 20 µM of arsenic trioxide (Trisenox) for 4 h then mitochondrial ROS production was assessed by flow cytometry using MitoSox. When indicated, cells were pre-treated with 10 mM NAC followed by incubation with 20 µM PEITC for 18 h or with 20 µM PEITC for 4 h. MitoSox fluorescence intensity was presented in arbitrary units (A.U.). Data are means of three independent experiments (SD<10%); (B) DA1-3b and DA1-3b/M2 cells were treated with PEITC for 18 h or Trisenox for 4 h in the presence or absence of pretreatment with the antioxidant NAC (10 mM for 1 h). Apoptotic cell death was then determined by flow cytometry after annexin V–FITC and propidium iodide staining. Flow cytometric profiles shown are representative of two replicate experiments; (C) Effect of loss of HIF-1α expression in DA-13b/M2 cells on ROS production and cell death. DA1-3b/M2 cells were treated with either HIF-1α siRNA or control non-targeting siRNA. At 24 h post transfection, cells were treated with 20 µM Trisenox for 4 h, 20 µM PEITC for 18 h or 300 µM Menadione for 1 h, then ROS production and cell death were determined by flow cytometry after MitoSox or PI staining, respectively. Results are presented as the percentage of inhibition of ROS production or cell death in siRNA HIF-1α transfected cells relative to siControl. (D) *In vivo* therapeutic activity of PEITC and Trisenox Survival of mice after injection of 1×10^6^ GFP-tagged DA1-3b/M2 cells. Cumulative survival data were compiled from two separate experiments and presented by a Kaplan–Meier survival analysis. GFP+ DA1-3b/M2 bearing mice were treated with Trisenox (4 mg/kg ip 5days/week) or PEITC (50 mg/kg ip 5days/week). Survival of mice treated with Trisenox (n = 10) or with PEITC (n = 10) in comparison to control animals (n = 20) was significant (P = 0.006 and P = 0.0008, respectively); (E) Decrease in percentage GFP+ DA1-3b/M2 cells in spleen after Trisenox and PEITC treatment. Untreated mice, PEITC- or Trisenox- treated mice were killed 37 days after inoculation of GFP+ DA1-3b/M2 cells. The percentage of GFP+ cells in spleen was evaluated by flow cytometry. Data are means +/− SD of three independent experiments; (F) Mitochondrial ROS levels in GFP-tagged DA1-3b/M2 cells after Trisenox and PEITC treatment *in vivo*. Untreated mice, PEITC- or Trisenox- treated mice were killed 37 days after inoculation of GFP+ DA1-3b/M2 cells. Spleen cells were stained with MitoSox to determine relative levels of ROS. The experiment was performed twice. Results are fold change in Mitosox fluorescence intensity of GFP^−^ and ^+^ spleen cells. The values for untreated cells were normalized to 1 for the analysis. As control, cells were treated with 100 µM menadione for 1 h *in vitro*.

We next tested if these mitochondrial alterations leading to ROS production could be therapeutically exploited to preferentially kill imatinib-resistant leukemic cells. As other imatinib-resistant cells [16], DA1-3b/M2, K562-IM, K562-NI cells produced more ROS when exposed to the pro-oxidants PEITC and arsenic trioxide (Trisenox™) than their sensitive counterparts (Fig. 6A and Fig. S3B). Imatinib-resistant cells were more prone to PEITC or Trisenox-induced apoptosis than imatinib sensitive cells as revealed by FACS analysis after Annexin V/PI staining (Fig. 6B and Fig. S3C). Pre-treatment of cells with NAC markedly reduced PEITC and Trisenox cytotoxicity as well as it prevented the generation of ROS (Fig. 6B and Fig. S3C) suggesting that increased ROS levels are responsible for the pro-apoptotic effects of PEITC and Trisenox in imatinib-resistant cells. Interestingly, both ROS production and cell death induced by pro-oxidants were reduced in DA1-3b/M2 cells when HIF-1α was knocked down (Fig. 6C).

To verify the relevance of the results *in vivo*, we investigated the antitumor effects of PEITC and Trisenox in a syngeneic leukemic model where injections of small GFP-tagged DA1-3b/M2 cell numbers led to an aggressive acute leukemia. Mice treated with PEITC or Trisenox lived significantly longer than untreated mice (Fig. 6D) in correlation with the lower percentage of GFP+ cells in spleen (Fig. 6E). To determine whether the antitumor effect was related to ROS production, analysis of spleen tissues following *in vivo* treatments showed that GFP+ cells produced higher levels of ROS compared to GFP cells, a situation similar to that observed after *ex vivo* menadione exposure (Fig. 6F).

## Discussion

Cumulative evidence suggests that alterations in glucose metabolism are associated with Bcr-Abl [7], [12], [34], [35]. These alterations include increased rates of cytosolic glycolysis along with reduction in the pentose phosphate cycle activity [35]. Our findings are compatible with the observation that Bcr-Abl positive cells greatly depend on glycolysis to supply ATP for proliferation [34]. We also found a modest but significant induction of genes encoding for glucose transporters and glycolytic enzymes in imatinib-resistant leukemic cells. Most of these genes are known to be under the control of HIF-1α [36], which has been found abnormally stabilized in imatinib-resistant leukemic cells ([35] and Fig. 1D). These data suggest that the coordinated induction of genes by HIF-1α may account for a part of the reprogramming of glucose from oxidation to glycolysis in imatimib-resistant leukemic cells.

In the last century, Otto Warburg formulated the hypothesis that metabolic modifications resulted from impairment of cancer cell mitochondria to undergo respiration [37]. Therefore, in attempting to understand the metabolic organization of imatinib-resistant cells, we focused our attention on mitochondria. Herein, we demonstrated that imatinib-resistant leukemic cells are characterized by mitochondrial alterations. An irreversible injury to cellular respiration has been proposed to explain the dependence of tumours on glycolytic energy [37]. Unlike the originally belief, our results indicate that increased glycolysis is not the result of an irremediable inability of mitochondria to utilize oxidative phosphorylation to produce ATP. In our experiments, glucose withdrawal does not lead to total ATP depletion in Bcr-Abl positive cells. This is accomplished by a shift from glycolysis to mitochondrial OXPHOS. In addition to enhanced glycolysis, our findings are compatible with the fact that the TCA cycle of Bcr-Abl positive cells could be fed by glutamate.

**Figure 7:**
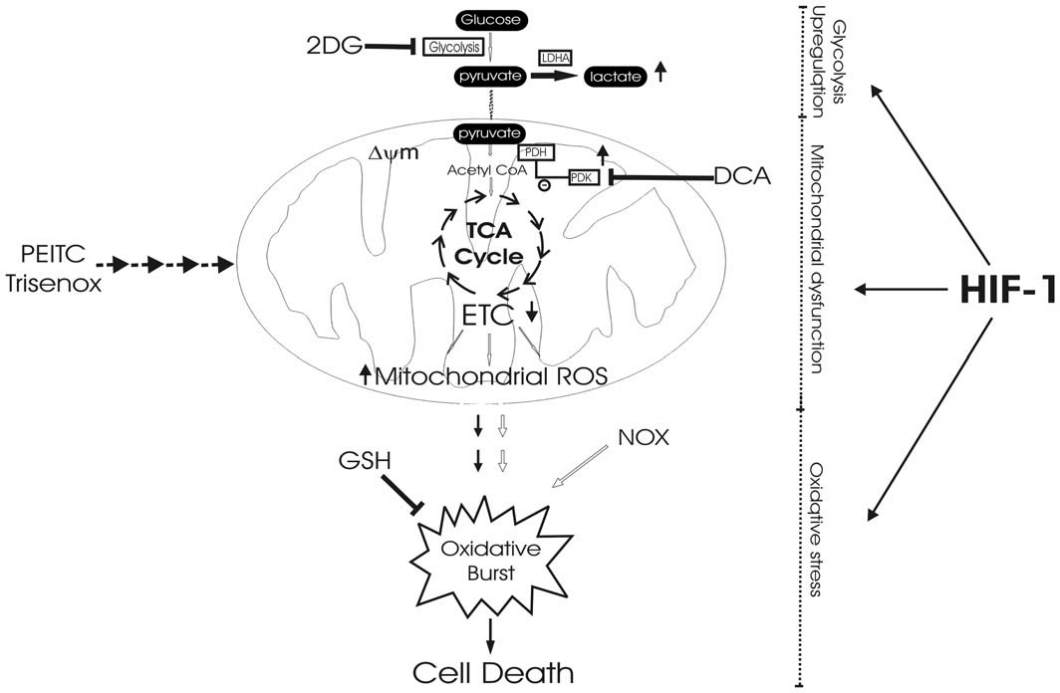
Suggested sequence of metabolic alterations leading to cell death in imatinib-resistant cells. Imatinib-resistant cells presented high glycolytic activity controlling mitochondrial oxidative phosphorylation. As a result, mitochondrial dysfunction generated more ROS spontaneously and after treatment with the pro-oxidants PEITC and Trisenox enhancing cell death sensitivity. Metabolic organization in imatinib-resistant cells is under control of HIF-1α (see text for details).

Besides, this study shows that glucose metabolism can exert a high degree of control over oxidative phosphorylation (Fig. 7). Use of 25 mM glucose is conformed to requirements reported for most of leukemic cell cultures but far higher than *in vivo* levels. Similar to results with high glucose medium, DA1-3b/M2 mitochondria were repressed when studied *in vitro* after incubation with lower glucose concentrations or recovered *ex vivo* from injected mice (not shown) indicating that mitochondrial dysfunction of DA1-3b/M2 cells is not merely a cell culture artefact. As pointed out by Sattler [13], [33], mitochondrial ROS are overproduced in Bcr-Abl positive cells. Our observations provide mechanistic insights to previous studies indicating suppression of glycolysis with 2-DG inhibited mitochondrial ROS generation [13]. The prevailing hypothesis raised to explain this effect on mitochondria was that high glycolysis leads to increased pyruvate oxidation that would stimulate mitochondrial respiratory chain and ROS overproduction as an unavailable by-product of increased electron transfer. According to this hypothesis, impairment of glucose metabolism (with 2-DG or galactose) would reduce pyruvate oxidation in mitochondria, thereby decreases mitochondrial respiration and ROS production. Although this has been examined in a number of cell types [38], we provide evidence that an alternative mechanism may apply in imatinib-resistant leukemic cells (Fig. 7). Indeed, we observed that inhibition of glycolysis causes derepression of respiration and that the subsequent increase in mitochondrial oxygen consumption limits intracellular oxygen leading to lower cellular ROS.

In tumours, entry of pyruvate into the TCA cycle is reduced by inhibition of pyruvate dehydrogenase (PDH) by pyruvate dehydrogenase kinase (PDK). Inhibition of PDH decreases mitochondrial ETC activity thereby diminishing ROS generation [39]. In imatinib-resistant cells, we observed a concomitant increase in PDK3 and a decrease in PDH shunting the pyruvate away from mitochondria, thereby resulting in the attenuation of mitochondrial respiration. Inhibition of PDK with DCA boosts mitochondrial activity and ROS generation in imatinib-sensitive and -resistant cells. However, the effect of DCA was significantly reduced in DA1-3/M2 cells suggesting that DCA cannot completely restore the activity of mitochondria in DA1-3b/M2 cells. Nevertheless, administration of DCA may constitute an alternative strategy to induce ROS-mediated apoptosis in Bcr-Abl positive cells. Irrespective of these considerations, impairment of mitochondrial function in imatinib-resistant cells results in accumulation of the TCA intermediates, succinate and fumarate. Accumulation of succinate and fumarate in cancer cells induces inhibition of HIF prolyl hydroxylases allowing the nuclear translocation of HIF-1α [40]. Therefore, our results suggest that the TCA cycle inhibition observed in imatinib-resistant cells may participate to the abnormal stabilization of HIF-1α [35].

In this report, we describe an association between mitochondrial dysfunction and imatinib resistance in various Bcr-abl expressing cell lines and in some CML patients with accelerated phase of CML. These results call for further investigation in additional patients to determine the exact association and the mechanism of mitochondrial dysfunction in imatinib resistance. Nevertheless, these mitochondrial alterations were most probably not due to BCR-ABL mutations since they are present in both non mutated (K562-IM, K562-NI [19], [20]) and mutated (DA13b/M2) imatinib-resistant cells. Why imatinib resistant cells adopt this metabolic profile? Glycolysis is essential for most transformed cells [41] providing an important growth advantage. Recent work showed that high glycolysis plays a key role in resistance to imatinib via p53 inactivation [42]. Additionally, mitochondrial dysfunction may also play a role in imatinib resistance via the production of mitochondrial ROS (Fig. 7). Indeed, it is noteworthy that oxidant stress participates to genomic instability of cancer cells by inducing oxidative DNA damage. In CML, ROS generation provokes mutations in kinases domain of Bcr-Abl contributing to emergence of resistant cells [43].

Finally, we show that these mitochondrial alterations leading to ROS overproduction can be exploited therapeutically to kill imatinib-resistant leukemic cells. Treatment strategies that focus on mitochondrial ROS production represent important opportunities against Bcr-Abl (+) cells [15], [44]. Herein, we demonstrated that the ROS-generating agents, PEITC or arsenic trioxide selectively killed imatinib-resistant cells through mitochondrial ROS-mediated damages. Overall, our results support the hypothesis that a coherent comprehension of the metabolic organization represents a new paradigm helpful to develop therapeutic strategies selectively designed to overcome imatinib resistance.

## Supporting Information

Figure S1 **The human imatinib resistant cell lines, K562-IM and K562-NI, express high levels of glycolytic enzymes.** (A) Quantitative PCR analysis of relative transcript levels of glycolytic enzymes and enzymes related to lactate metabolism in K562-IM and K562-NI cells compared with K562 cells. Data are means +/− SD of three independent experiments; (B) Western blots analysis and quantification of several glycolytic-related proteins in total lysates of K562 and K562-IM, K562-NI cells. Actin protein amounts are used to check equal loading of proteins. Four independent immunoblottings were scanned on a densitometer and the expression of proteins in K562-IM or K562-NI was determined relative to expression in K562 after normalization with αctin. The mean intensity of the values obtained was expressed in arbitrary units.
(TIF)

Figure S2 **Evidence of mitochondrial dysfunction in the human imatinib resistant cell lines, K562-IM and K562-NI.** (A) Flow cytometric determination of ΔΨ_m_ using JC-1 staining. Results are expressed as in Figure 2C. Data are means+/− SD of three independent experiments made in duplicates; (B) Representative oxygen consumption tracings of K562, K562-IM and K562-NI cells. When compared to the human imatinib-sensitive cell line K562, K562-IM or K562-NI cells demonstrated a pronounced reduction in respiration. Data are representative of three independent experiments; (C) Proportions of mitochondrial oxygen consumption due to proton leak and ATP turnover in K562, K562-IM and K562-NI cells.
(TIF)

Figure S3 **PEITC and Trisenox induce overproduction of mitochondrial ROS and subsequent cell death in the human imatinib resistant cell lines, K562-IM and K562-NI.** (A) Cytofluorometric analysis of mitochondrial ROS production in K562, K562-IM and K562-NI cells kept untreated or incubated with menadione (100 µM, 1 h) in the presence or absence of 10 mM NAC. MitoSox fluorescence intensity was presented in arbitrary units (A.U.) Data are means of 3 independent experiments; (B) Cytofluorometric analysis of mitochondrial ROS production in K562, K562-IM and K562-NI cells treated with the indicated doses of PEITC or Trisenox. MitoSox fluorescence intensity was presented in arbitrary units (A.U.). Data are means of 2 independent experiments; (C) Flow cytometric profiles of PEITC (50 µM for 18 h) and Trisenox (20 µM for 18 h)-induced apoptosis in K562, K562-IM and K562-NI cells using Annexin V-FITC and PI staining. NAC (10 mM) was used to confirm the role of ROS in PEITC- and Trisenox-induced cell death. Data are representative of three independent experiments.
(TIF)

Table S1 **Characteristics of imatinib-resistant CML patients included in this study.**
(DOC)

Material and Methods S1 **List of primers used in this study.**
(DOC)
