# Supplementary figures and images for: Exploiting Mitochondrial Dysfunction for Effective Elimination of Imatinib-Resistant Leukemic Cells
Source: PLoS One. 2011 Jul 18;6(7):e21924. doi: 10.1371/journal.pone.0021924 (PMC3138741; doi:10.1371/journal.pone.0021924)

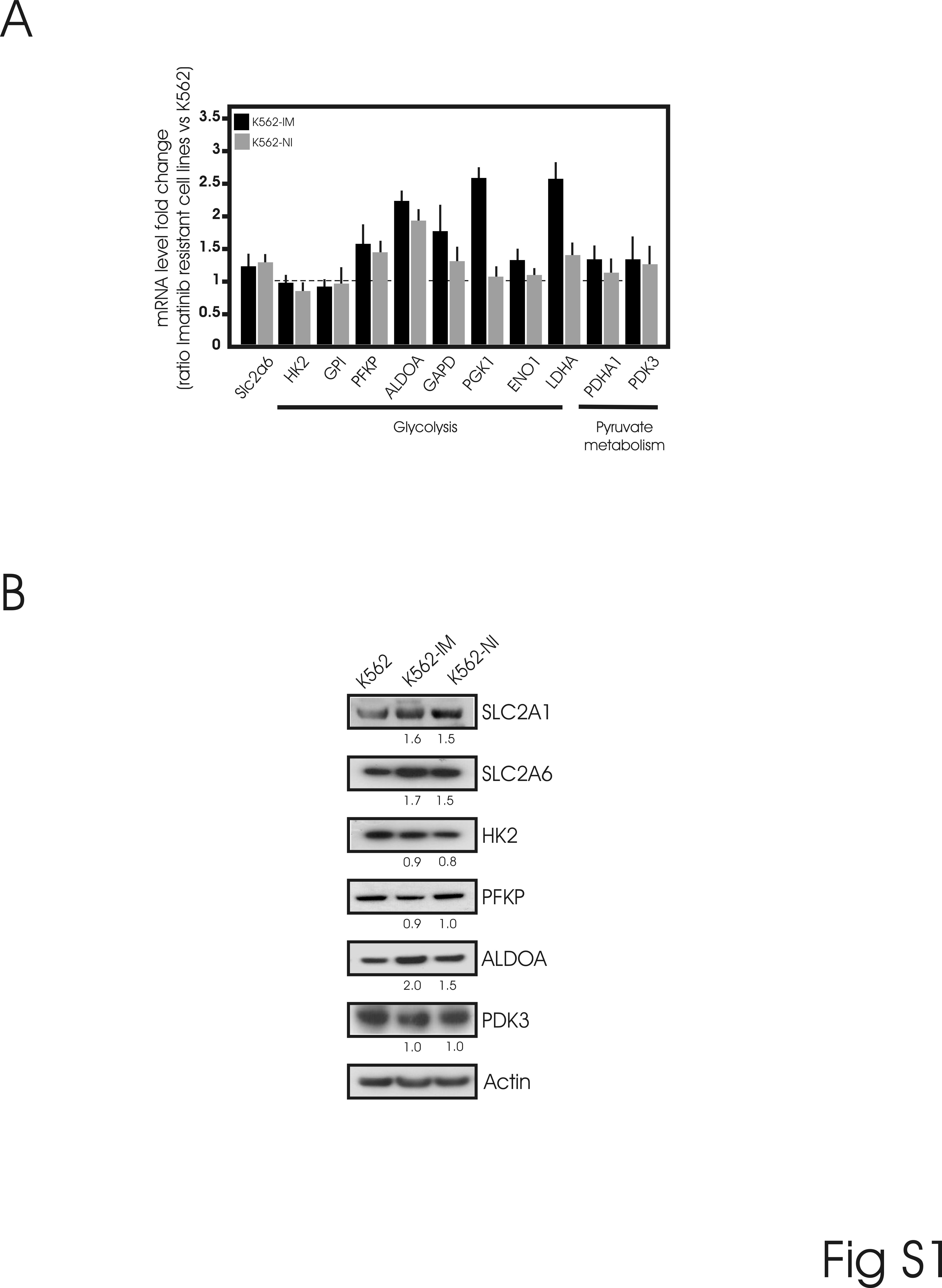

Supplement: Figure S1 — The human imatinib resistant cell lines, K562-IM and K562-NI, express high levels of glycolytic enzymes. (A) Quantitative PCR analysis of relative transcript levels of glycolytic enzymes and enzymes related to lactate metabolism in K562-IM and K562-NI cells compared with K562 cells. Data are means +/− SD of three independent experiments; (B) Western blots analysis and quantification of several glycolytic-related proteins in total lysates of K562 and K562-IM, K562-NI cells. Actin protein amounts are used to check equal loading of proteins. Four independent immunoblottings were scanned on a densitometer and the expression of proteins in K562-IM or K562-NI was determined relative to expression in K562 after normalization with αctin. The mean intensity of the values obtained was expressed in arbitrary units. (TIF) [file pone.0021924.s001.tif]

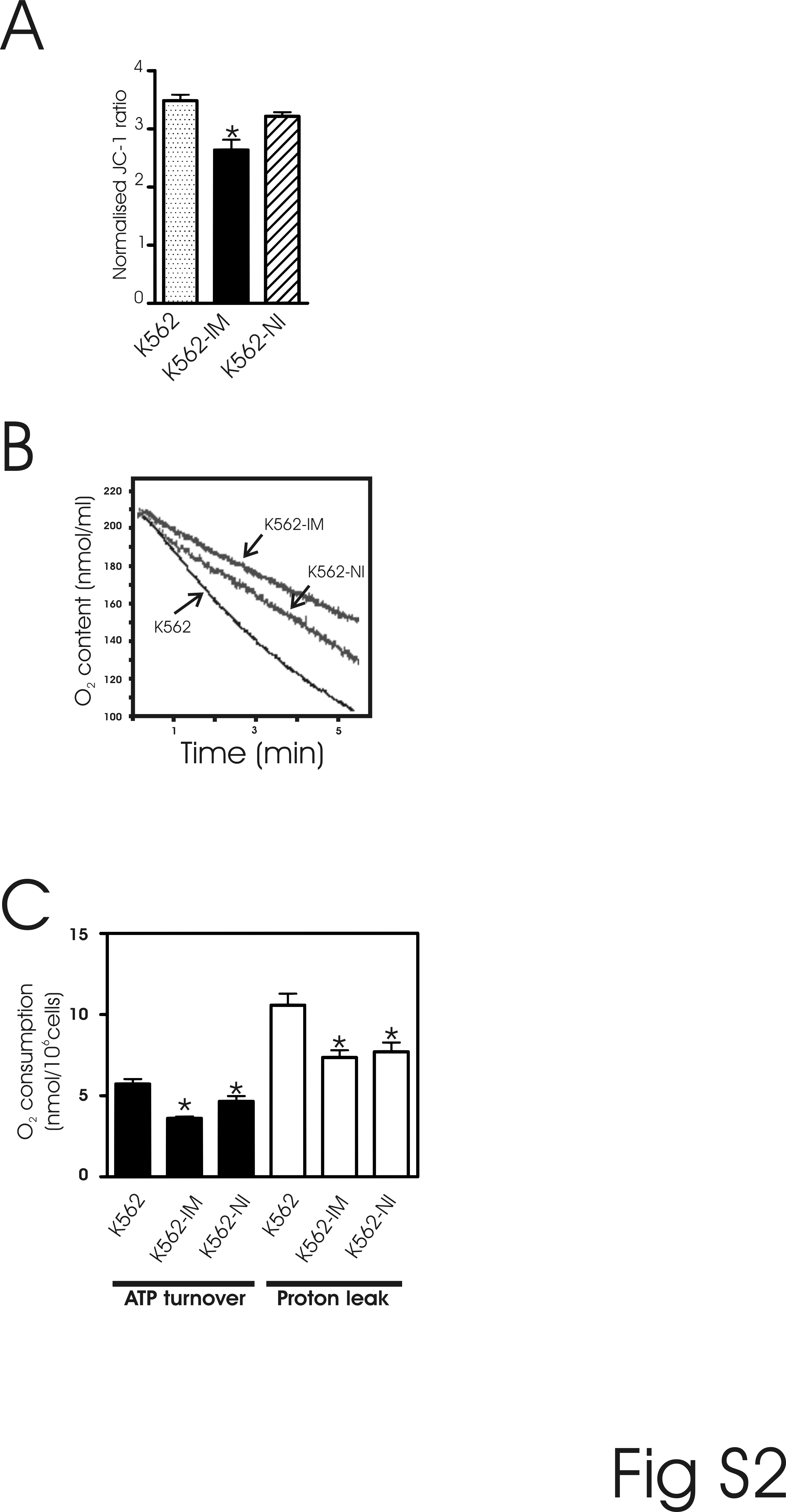

Supplement: Figure S2 — Evidence of mitochondrial dysfunction in the human imatinib resistant cell lines, K562-IM and K562-NI. (A) Flow cytometric determination of ΔΨm using JC-1 staining. Results are expressed as in Figure 2C. Data are means+/− SD of three independent experiments made in duplicates; (B) Representative oxygen consumption tracings of K562, K562-IM and K562-NI cells. When compared to the human imatinib-sensitive cell line K562, K562-IM or K562-NI cells demonstrated a pronounced reduction in respiration. Data are representative of three independent experiments; (C) Proportions of mitochondrial oxygen consumption due to proton leak and ATP turnover in K562, K562-IM and K562-NI cells. (TIF) [file pone.0021924.s002.tif]

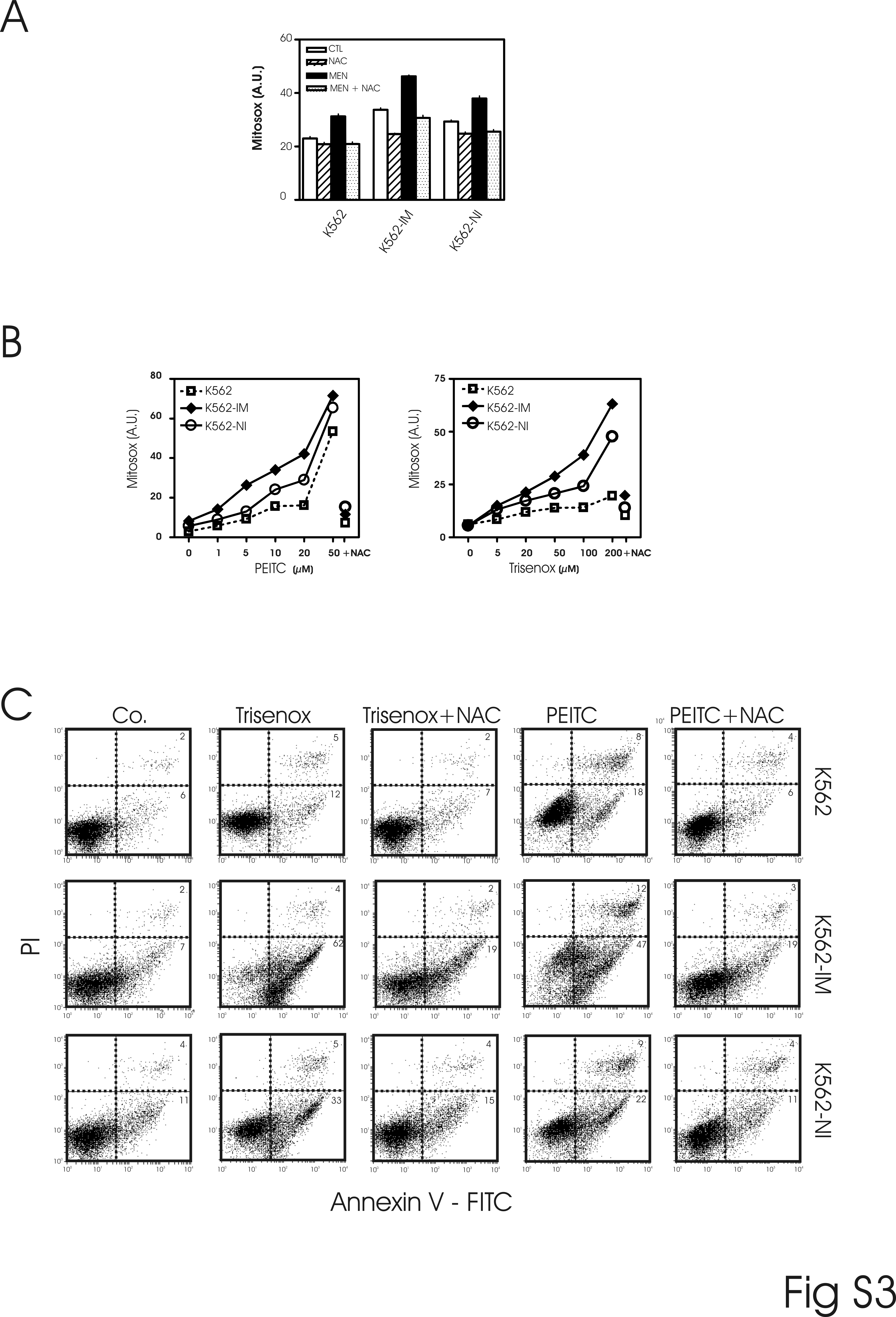

Supplement: Figure S3 — PEITC and Trisenox induce overproduction of mitochondrial ROS and subsequent cell death in the human imatinib resistant cell lines, K562-IM and K562-NI. (A) Cytofluorometric analysis of mitochondrial ROS production in K562, K562-IM and K562-NI cells kept untreated or incubated with menadione (100 µM, 1 h) in the presence or absence of 10 mM NAC. MitoSox fluorescence intensity was presented in arbitrary units (A.U.) Data are means of 3 independent experiments; (B) Cytofluorometric analysis of mitochondrial ROS production in K562, K562-IM and K562-NI cells treated with the indicated doses of PEITC or Trisenox. MitoSox fluorescence intensity was presented in arbitrary units (A.U.). Data are means of 2 independent experiments; (C) Flow cytometric profiles of PEITC (50 µM for 18 h) and Trisenox (20 µM for 18 h)-induced apoptosis in K562, K562-IM and K562-NI cells using Annexin V-FITC and PI staining. NAC (10 mM) was used to confirm the role of ROS in PEITC- and Trisenox-induced cell death. Data are representative of three independent experiments. (TIF) [file pone.0021924.s003.tif]
