# Supplementary material for: Exploiting Mitochondrial Dysfunction for Effective Elimination of Imatinib-Resistant Leukemic Cells
Source: PLoS One. 2011 Jul 18;6(7):e21924. doi: 10.1371/journal.pone.0021924 (PMC3138741; doi:10.1371/journal.pone.0021924)
Supplement: Table S1 — Characteristics of imatinib-resistant CML patients included in this study. (DOC) [file pone.0021924.s004.doc]

Supporting Table S1: Characteristics of imatinib-resistant CML patients

| CML patient | **Imatinib resistance** | **Phase of the disease** | **Karyotype** |
| --- | --- | --- | --- |
| **Patient 1** | Primary resistance | Chronic Phase | del(7)p,t(9 ;22) |
| **Patient 2** | Partial response | Chronic Phase | t(9;22) |
| **Patient 3** | + | Blast Crisis | t(9 ;22)+8+12+21 add(17)(p11)add(5)(q32)add(11)(p11)del(14)(q22) |
| **Patient 4** | + | Blast Crisis | t(9 ;22), inv(3)(q21,q26), der(13)t(8 ;13)(p11 ;p11) 10 |
