# Supplementary material for: Exploiting Mitochondrial Dysfunction for Effective Elimination of Imatinib-Resistant Leukemic Cells
Source: PLoS One. 2011 Jul 18;6(7):e21924. doi: 10.1371/journal.pone.0021924 (PMC3138741; doi:10.1371/journal.pone.0021924)
Supplement: Material and Methods S1 — List of primers used in this study. (DOC) [file pone.0021924.s005.doc]

**Material and Methods S1: List of primers used in this study :**

The sequences of mouse PCR primers used are (all are listed 5’ to 3’):SCLC2A1 forward GATTGGCTCCTTCTCTGTGG/ SCLC2A1 reverse TCAAAGGACTTGCCCAGTTT; SLC2A3 forward TCTCAGCAGCTCTCTGGGAT/ SLC2A3 reverse ATCGTGG CATAGATCGGTTC; SLC2A6 forward TTGGATC CTGACCTGCATCT/SLC2A6 reverse GAGGTCGTTGAGGATCATGG; HK2 forward AACCATGACCAAGTGCAGAA/ HK2 reverse AGCCCTTTCTCCATCTCCTT; GPI forward AAACATGTTCGAGTTCTGGGA/ GPI reverse GCTCGAAGTTGTCAAAACCC; PFKP forward GGGCCAAGGTGTA CTTCATC/ PFKP reverse TGGAGACACTCTCCCAGTCG; ALDOA forward CTCTCCT TGAATCCACTCGC/ ALDOA reverse TAGGGCATGGTGCTGGTAGT; GAPD forward AAGGTGAAGGTCGGAGTCAA/ GAPD reverse AATGAAGGGGTCATTGATGG; PGK1 forward CAAGCTGGACGTTAAAGGGA/ PGK1 reverse CTTGGGACAGCAGCCTTAAT; ENO1 forward GCCTCCTGCTCAAAGTCAAC/ ENO1 reverse AACGATGAGAC ACCATGACG; PKM2 forward TCCGGATCTCTTCGTCTTTG/ PKM2 reverse GTCTGAATGAAGGCAGTCCC; LDHA forward GGCCTGTGCCATCAGTATCT/ LDHA reverse GGAGATCCATCATCTCTCCC; PDHA1 forward TCATGAGGAAGATGCTCGC /PDHA1 reverse TTTGCAAAATTACGGGATGC; PDK3 forward TCTCCATCAAACAATTCCTGG/ PDK3 reverse TCTCATTGTGTTAGCCAGCC; 18s reverse CGGACAGGATTGACAGATTG/18s forward CAAATCGCTCCACCAACTAA.

The sequences of human PCR primers used are (all are listed 5’ to 3’): 18s forward TAGAGGGACAAGTGGCGTTC/ reverse CGCTGAGCCAGTCAGTGT'; ALDOA forward CTCTCCTTGAATCCACTCGC/ reverse TAGGGCATGGTGCTGGTAGT; ENO1 forward GCCTCCTGCTCAAAGTCAAC/ reverse AACGATGAGACACCATGACG; GPI1 forward AAACATGTTCGAGTTCTGGGA/ reverse GCTCGAAGTTGTCAAAACCC; GAPDH Forward AAGGTGAAGGTCGGAGTCAA/ reverse AATGAAGGGGTCATTGATGG; HK2 forward AACCATGACCAAGTGCAGAA/ reverse AGCCCTTTCTCCATCTCCTT; LDHA forward ATGGCCTGTGCCATCAGTAT/ reverse TTCTAAGGAAAAGGCTGCCA, PDHA1 forward TCATGAGGAAGATGCTCGC/ reverse TTTGCAAAATTACGGGATGC; PDK3 forward TCTCCATCAAACAATTCCTGG/ reverse TCTCATTGTGTTAGCCAGCC; PFKP forward GGGCCAAGGTGTACTTCATC/ reverse TGGAGACACTCTCCCAGTCG; PGK1 forward CAAGCTGGACGTTAAAGGGA/ reverse CTTGGGACAGCAGCCTTAAT; SLC2A6 forward TTGGATCCTGACCTGCATCT/ reverse GAGGTCGTTGAGGATCATGG.
